# Photochemical Synthesis of Silver Hydrosol Stabilized by Carbonate Ions and Study of Its Bactericidal Impact on *Escherichia coli*: Direct and Indirect Effects

**DOI:** 10.3390/ijms23020949

**Published:** 2022-01-16

**Authors:** Vadim Ershov, Natalia Tarasova, Evgeny Abkhalimov, Alexey Safonov, Vladimir Sorokin, Boris Ershov

**Affiliations:** 1 Frumkin Institute of Physical Chemistry and Electrochemistry, Russian Academy of Sciences, 119071 Moscow, Russia; vadersh@yandex.ru (V.E.); abkhalimov@ipc.rssi.ru (E.A.); alexeysafonof@gmail.com (A.S.); 2 Institute of Chemistry and Problems of Sustainable Development, Dmitry Mendeleev University of Chemical Technology of Russia, 125047 Moscow, Russia; tarasnp@muctr.ru; 3 Winogradsky Institute of Microbiology, Research Center of Biotechnology of the Russian Academy of Sciences, 119071 Moscow, Russia; vlvlsorokin@gmail.com

**Keywords:** nanoparticles, dissolution, antibacterial, toxicity, reactive oxygen species, oxygen stress

## Abstract

The great attention paid to silver nanoparticles is largely related to their antibacterial and antiviral effects and their possible use as efficient biocidal agents. Silver nanoparticles are being widely introduced into various areas of life, including industry, medicine, and agriculture. This leads to their spreading and entering the environment, which generates the potential risk of toxic effect on humans and other biological organisms. Proposed paper describes the preparation of silver hydrosols containing spherical metal nanoparticles by photochemical reduction of Ag^+^ ions with oxalate ions. In deaerated solutions, this gives ~10 nm particles, while in aerated solutions, ~20 nm particles with inclusion of the oxide Ag_2_O are obtained. Nanoparticles inhibit the bacterium *Escherichia coli* and suppress the cell growth at concentrations of ~1 × 10^−6^–1 × 10^−4^ mol L^−1^. Silver particles cause the loss of pili and deformation and destruction of cell membranes. A mechanism of antibacterial action was proposed, taking into account indirect suppressing action of Ag^+^ ions released upon the oxidative metal dissolution and direct (contact) action of nanoparticles on bacterial cells, resulting in a change in the shape and destruction of the bacteria.

## 1. Introduction

The synthesis and applications of nanosized metal particles is currently one of the most rapidly developing fields of nanotechnologies [1,2,3,4,5,6]. Silver nanoparticles of various shapes and sizes occupy a special place among them [7,8,9]. Like other nanoscale metals, they have a large surface area to volume ratio, which endows them with unique properties and accounts for high efficiency of their action. Silver nanoparticles are known to exhibit relatively high biocidal and fungicidal activities [10,11,12]; therefore, one of numerous applications of silver nanoparticles is medicine. In view of the increase in the bacterial resistance to the existing antibiotic drugs, the search for alternative agents for fast and efficient combating of pathogenic microbial strains is highly relevant. In this respect, a promising approach is functionalization of silver nanoparticle surface, which enhances their biocidal activity [13,14,15].

The mechanism of action of silver on microorganisms is mainly associated with adsorption of silver ions [16]. Cells lose viability as a result of electrostatic interaction with positively charged silver ions adsorbed by a negatively charged bacterial cell. Silver ions are bound by the outer membrane peptidoglycans, thus blocking their ability to transport oxygen into the cell. This leads to cell suffocation and death of the microorganism [17].

The action of silver nanoparticles was interpreted in terms of redox mechanisms involving free radicals, resulting from oxidation of the nanoparticle surface. Vishnupriya et al. [18] demonstrated that when entering the *Escherichia coli* (*E. coli*) cell, silver nanoparticles mainly interact with proteins and DNA, thus damaging them. Kim et al. [19] found that the growth of *E. coli* can be inhibited due to the formation of free radicals on the silver nanoparticle surface, which may induce damage of the membrane. Kim et al. [20] confirmed these results by demonstrating that the antibacterial activity of silver nanoparticles against *E. coli* depends on the reactive oxygen species (ROS), which induce protein cleavage due to enhanced permeability in the membrane and enzyme inactivation.

A key aspect related to the medicinal application of nanoparticles is the necessity to ensure the particle stability. In the synthesis of nanoparticles, non-toxic compounds that preserve the main functional properties of silver should be used as reducing agents and hydrosol stabilizing agents. Therefore, it is important to prepare stable hydrosols composed of nanoparticles of moderate size containing no toxic impurities and stable in biological media. Previously, we developed a method for the synthesis of stable silver particles of moderate size complying with the green chemistry principles [21,22,23,24]. The method includes UV-induced reduction of Ag^+^ ions with C_2_O_4_^2−^ oxalate ions. This gives metal nanoparticles stabilized by carbonate ions formed upon degradation of the oxalate. This procedure affords hydrosols containing almost only silver nanoparticles and carbonate anions. The salt composition of these solutions mimics that of natural freshwater.

The purpose of the present study is to evaluate the antibacterial action of silver NPs obtained by photochemical reduction of Ag^+^ ions with oxalate ions on the bacterial cells of *Escherichia coli* K-12 MG1655. A specific feature of the study is the use of “pure” hydrosol containing no polymeric stabilizers, reducing agents, or their decomposition products, which are almost inevitably present when traditional methods for the preparation of silver nanoparticles are used. This is an important circumstance that eliminates the interference of toxic reducing and stabilizing agents in the evaluation of the antibacterial properties of silver. Additionally, we compared the effect of silver nanoparticles of ~10 nm size obtained in deaerated conditions with that of oxidized silver nanoparticles of ~20 nm size prepared in aerated conditions.

## 2. Results

### 2.1. Characterization of Silver Nanoparticles

**Figure 1:**
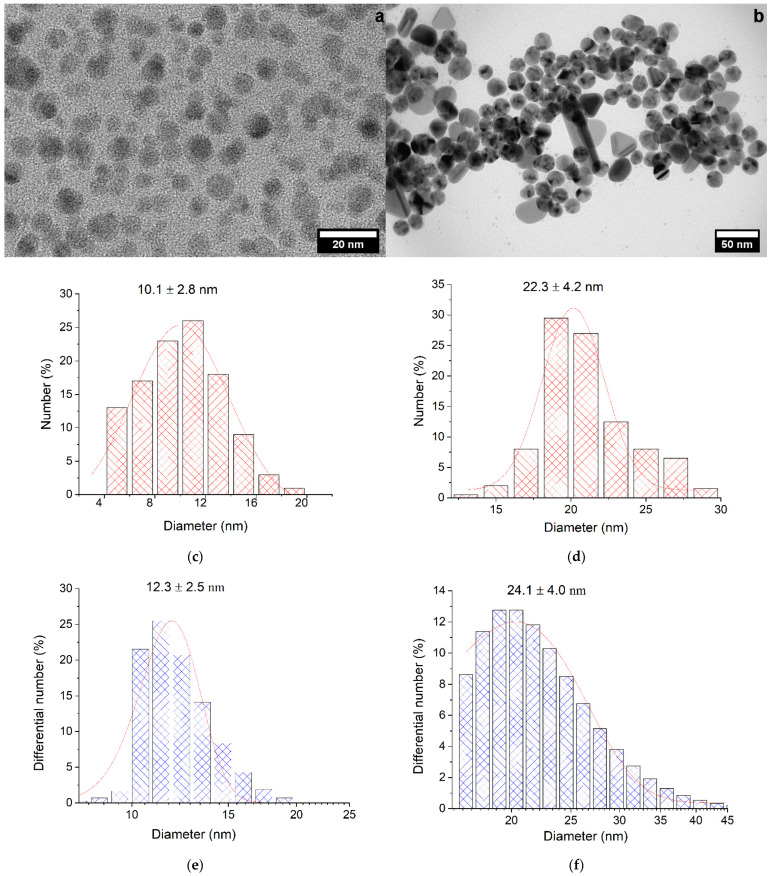
TEM and DLS images of silver nanoparticles and particle-size-distribution histograms. Solution: [Ag^+^] = 3 × 10^−4^ mol L^−1^; [C_2_O_4_^2−^] = 5 × 10^−4^ mol L^−1^; (**a**,**c**,**e**) deaerated; (**b**,**d**,**f**) in air. pH = 7.0 ± 0.1.

The photochemical reduction of Ag^+^ ions with oxalate ions both in the presence and in the absence of air oxygen gave spherical nanoparticles (Figure 1a,b) with a narrow size distribution. The average size of NPs obtained in the absence of air calculated from TEM data were 10.1 ± 2.8 nm (Figure 1c) (these nanoparticles will be designated as Ag-NPs), while the size of NPs formed in the presence of air oxygen was 22.3 ± 4.2 nm (Figure 1d) (they will be designated as Ag-Ag_2_O-NPs). The size of the silver colloid, including the stabilizing electrical double layer (EDL), was measured by DLS and amounted to 12.3 ± 2.5 nm for NPs obtained in the absence of air (Figure 1e) and 24.1 ± 4.0 nm for particles obtained in air (Figure 1f). The slight difference between the sizes measured by TEM and DLS attests to a small size of the stabilizing layer.

**Figure 2:**
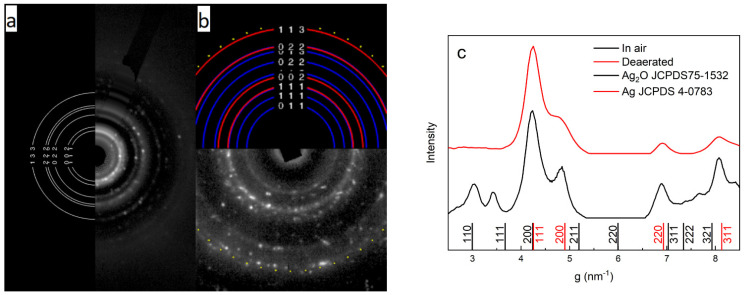
Diffraction pattern of Ag (**a**) and Ag_2_O-Ag (**b**) nanoparticles and comparison of the intensity of electron diffraction reflexes and JCPDS data (**c**), respectively.

According to electron diffraction analysis, the nanoparticles had a polycrystalline structure. The electron diffraction pattern of the nanoparticles obtained in air showed rings that indicate the presence of silver oxide (Ag_2_O), apart from the rings that refer to metallic silver (Figure 2).

The mechanism of photochemical formation of silver nanoparticles includes decomposition of oxalate ions to give CO_2_^−•^ radical ions with high reduction potential (*E*^0^(CO_2_/CO_2_^−•^) = −1.9 V) [25].

(CO_2_)_2_^2−^ → 2CO_2_^−•^ (1)

These radical ions efficiently reduce silver ions.

Ag^+^ + CO_2_^−•^ → Ag^0^ + CO_2_       *k* = 4.0 × 10^9^ mol L^−1^ s^−1^ (2)

The atoms agglomerate to give the metal:

nAg^0^ → Ag_n_ (3)

Carbon dioxide is transformed in water into carbonic acid, serving as the source of bicarbonate and carbonate ions, which stabilize the arising colloidal silver particles

CO_2_ + H_2_O ↔ H^+^ + HCO_3_^−^ ↔ 2H^+^ + CO_3_^2−^ (4)

In aerated solution, the CO_2_^−•^ radical ion reacts with oxygen to form reactive radical ion O_2_^−•^

CO_2_^−•^ + O_2_ → CO_2_ + O_2_^−•^       *k* = 2.4 × 10^9^ mol L^−1^ s^−1^ (5)

Reactive oxygen in the form of O_2_^−•^ also oxidizes silver atoms. Thus, in aerated solutions, agglomeration of silver atoms competes with their oxidation by O_2_ and O_2_^−•^. Apparently, the products of oxidation (Ag_2_O) are incorporated into the lattice and attached to the surface of forming nanoparticles.

**Figure 3:**
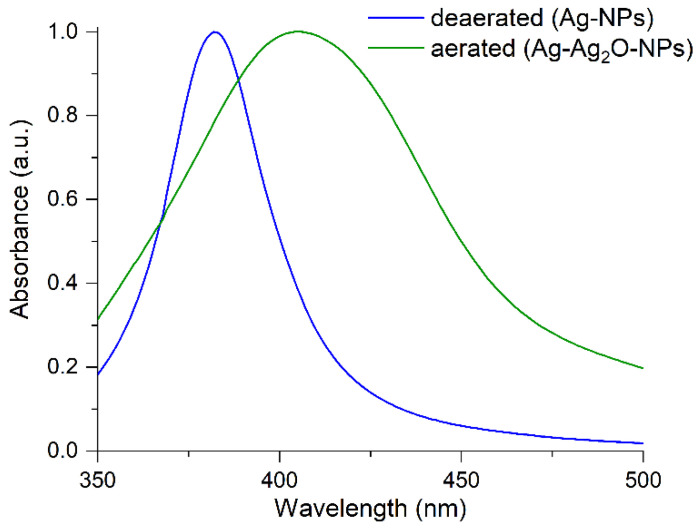
Absorbance of silver nanoparticles obtained in deaerated (Ag-NPs) and in aerated (Ag-Ag_2_O-NPs) solutions.

The presence of silver oxide changes the electronic state of silver nanoparticles. This is clearly seen upon comparison of the absorption spectra of localized surface plasmon resonance (LSPR) of particles obtained in deaerated (Ag-NPs) and aerated (Ag-Ag_2_O-NPs) conditions (Figure 3). The LSPR band of Ag-Ag_2_O-NPs is much wider and is shifted by 24 nm to longer wavelengths in comparison with that of the Ag-NPs. According to Mie-Drude theory [26,27], the shift of the LSPR band indicates that the electron density (concentration of conduction electrons) is lower in an Ag-Ag_2_O-NPs than in an Ag-NP by approximately 10–12%. A similar effect of Ag_2_O on the plasmon absorption was demonstrated for the oxidation of a monolayer of silver nanoparticles [28]. When the oxide was present on the surface, the LPSR band shifted to longer wavelengths (up to 440 nm). It should be noted that the thickness of the oxide on the silver surface can reach 2 nm and does not depend on the nanoparticle size.

The *ζ*-potentials of silver nanoparticles measured by the DLS method were −68.3 and −67.4 mV for Ag-NPs and Ag-Ag_2_O-NPs, respectively. The negative sign of the potential indicates that the EDL potential-forming ions of the colloids are anions (at pH 4–8, these are mainly bicarbonate ions HCO_3_^−^). Large absolute values of the potentials attest to high aggregation stability of the colloids. Indeed, hydrosols are stable for several months. The hydrosol stability increases with decreasing amount of the colloidal metal in the solution. Ag-Ag_2_O-NPs are much more stable than Ag-NPs. Table 1 summarizes characteristics of silver nanoparticles synthesized and used in the present study.

### 2.2. Stability of Silver Hydrosol in Culture Media

Silver is thermodynamically unstable in aqueous medium, and in the presence of air, its oxidative dissolution takes place [29]. In the absence of air, there is no dissolution of silver nanoparticles or release of silver ions to the solution [30,31,32,33]. The rate of dissolution depends on the nanoparticle size (the smaller the size, the higher the rate), pH (the rate increases upon acidification), the nature of other components present in the solution (compounds that form poorly soluble and/or stable components with Ag^+^ ion, such as OH^−^, Cl^−^, Br^−^, I^−^, S^2−^, and other ions accelerate the dissolution), and the ionic strength of the solution (the presence of inert salts accelerates the dissolution). Conversely, some organic stabilizing agents inhibit the oxidative dissolution, i.e., dissolution slows down. A temperature rise, illumination, and other factors increase the silver dissolution rate.

The oxidation of silver nanoparticles in water involves the oxide formation step [30]. The mechanism is apparently electrochemical and can be described by the following electrode half-reactions. At the anode, silver atoms of the nanoparticles give off electrons, and the resulting ions form the oxide phase (hereinafter, the standard electrode potentials are given according to [34]).

2Ag + 2OH^−^− 2e^−^ ↔ 2AgOH (Ag_2_O + H_2_O) *E*^0^_1_ = 0.342 V (6)

At the cathode, excess electrons interact with the dissolved oxygen, which is thus reduced

O_2_ + 2H_2_O + 4e^−^ ↔ 4OH^−^       *E*^0^_2_ = 0.401 V (7)

The non-oxidized silver surface cannot serve as a source of Ag^+^ ions [30]. They appear upon dissolution of the formed silver oxide

Ag_2_O + 2H^+^ → 2Ag^+^ + H_2_O (8)

According to [35], the Ag^+^ ions are released to the solution upon the dissolution of one or two oxidized monolayers from the nanoparticle surface.

The dissolution efficiency depends on the electromotive force (∆*E*^0^). ∆*E*^0^ for the oxidation of silver is equal to the difference between the standard potentials of reactions (6) and (7), i.e., ∆*E*^0^ = (*E*^0^_2_ − *E*^0^_1_) = 0.059 V. The positive ∆*E*^0^ value indicates that the redox process preferably proceeds toward metal oxidation; this is actually the case. The greater ∆*E*^0^, the higher the silver oxidation rate is.

Silver hydrosols stabilized by carbonate ions are relatively stable in pure water and in solutions resembling natural freshwater in the composition. The time it takes for the concentration of atomic silver in the hydrosol to decrease twofold (*τ*_1/2_) is approximately 2 to 3 months for Ag-NPs and 6 months for Ag-Ag_2_O-NPs.

**Figure 4:**
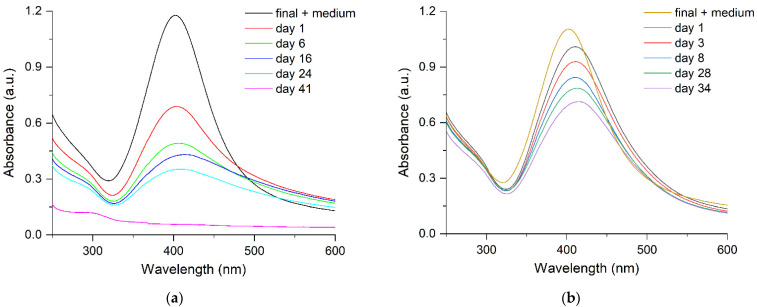
Absorption spectra of silver hydrosol (**a**) Ag-NPs and (**b**) Ag-Ag_2_O-NPs in modified liquid culture medium “Adkins M”.

**Figure 5:**
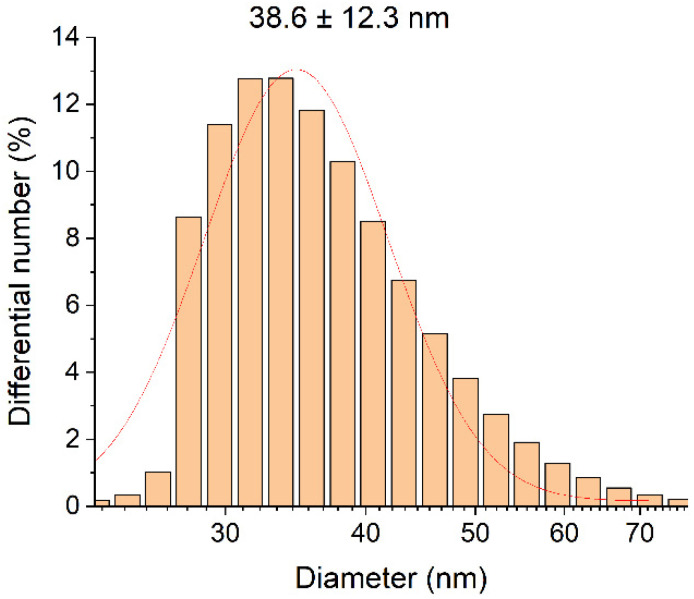
DLS diagrams of silver hydrosol Ag-Ag_2_O-NPs (20 nm) in liquid culture medium “Adkins M”.

An important factor for studying the antibacterial action of silver on the cells of *Escherichia coli* is the hydrosol stability in the culture medium used. The stability of hydrosols is usually much lower in culture media than in water. Indeed, according to the optical spectroscopy data (Figure 4a), the time *τ*_1/2_ of the Ag-NP hydrosol (10 nm) is approximately 3 days in the modified liquid Adkins culture medium (Adkins M) versus 2–3 months characteristic of pure water. Conversely, Ag-Ag_2_O-NPs (20 nm) show a markedly higher stability, with *τ*_1/2_ being 6–7 days. Within approximately 10–15 days, the structure of the optical LPSR band is clearly manifested, which is indicative of the nanostructured state and retention of the aggregative stability (Figure 4b). However, this is far shorter than 6 months, for which the hydrosol is stable in water. Please note that regarding the time of stability, both types of hydrosols are suitable for conducting expreriments on studying the antibacterial activity of silver on the *Escherichia coli* cells (48 h). The results of optical detection of the dissolution of silver nanoparticles in the Adkins M culture medium by monitoring the decrease in intensity of the interband electronic transition at 250 nm, which reflects the Ag^0^ concentration in the nanoparticle [22], are in good agreement with the results of analysis of Ag^+^ ions released to the solution. Indeed, after 24 and 48 h, the concentration of the dissolved metal in 1 × 10^−4^ mol L^−1^ Ag^0^ was approximately 14 and 25%, respectively, according to the results of optical measurement and 13 and 24.3%, respectively, according to IPC determination of Ag^+^ ions. The DLS data demonstrate that components of the Adkins M medium change the state of hydrosol. The size of silver aggregates was found to considerably (almost twofold) increase (Figure 5), which attests to agglomeration of silver colloids.

The decrease in the hydrosol stability in the presence of components of the culture medium is due to the fact that they normally contain considerable amounts of salts and organic compounds, which disrupt the composition and structure of the EDL and, hence, the stability of the colloid solution. For example, these are Cl^−^, Br^−^, I^−^, S^2−^, and some other ions, which form poorly soluble (AgCl) and even virtually insoluble (Ag_2_S) salts with Ag^+^. That is why the salt formation reactions presented below become preferable over reaction (6) giving Ag_2_O

AgCl + e^−^ ↔ Ag + Cl^−^      *E*^0^ = 0.222 V (9)

AgBr + e^−^ ↔ Ag + Br^−^      *E*^0^ = 0.071 V (10)

AgI + e^−^ ↔ Ag + I^−^       *E*^0^ = −0.152 V (11)

Ag_2_S + 2e^−^ ↔ 2 Ag + S^2−^     *E*^0^ = −0.691 V (12)

Consequently, the electromotive force (∆*E*^0^) of silver oxidation with participation of reactions indicated above is 0.179, 0.33, 0.553, and 1.092 V, respectively. In other words, ∆*E*^0^ is much higher than the value of 0.059 V for reaction (6) giving Ag_2_O. This series of increasing ∆*E*^0^ coincides with the order of increasing rate of oxidation of silver nanoparticles, i.e., decreasing stability of colloidal silver. The release of silver ions to the solution is determined by the reactions of poorly soluble salt phases with H^+^, halide ions, and other components to give Ag^+^ and soluble complexes such as AgCl_2_^−^, AgBr_2_^−^, and other. Thus, the complex composition of culture media and increase in the ionic strength of the solution in the presence of salts are responsible for the sharp decrease in the stability of hydrosols and promote aggregation of colloids. Higher stability of Ag-Ag_2_O-NPs (20 nm) compared to Ag-NPs (10 nm) is apparently due to the fact that they have a protective layer of the Ag_2_O oxide.

### 2.3. Antibacterial (Toxic) Action of Silver on the Bacterial Cells of Escherichia coli

The extensive use of silver is caused by its high biocidal activity, the ability to suppress harmful microflora. Meanwhile, the spread of silver nanoparticles in the environment may have an adverse effect on natural microbial communities. Entering the aquatic environment, which is the most probable destination upon diffuse scattering, is hazardous for living organisms and generates a real environmental threat [36,37,38]. To estimate the risk and to predict the possible environmental hazards, it is important to elucidate the characteristic features of these processes [39,40]. The Ag^+^ ions [41,42] and silver nanoparticles [43,44,45] are the main cause of toxicity of silver in the natural environment.

#### 2.3.1. Inhibition of Growth of *Escherichia coli* Bacterial Cells

**Figure 6:**
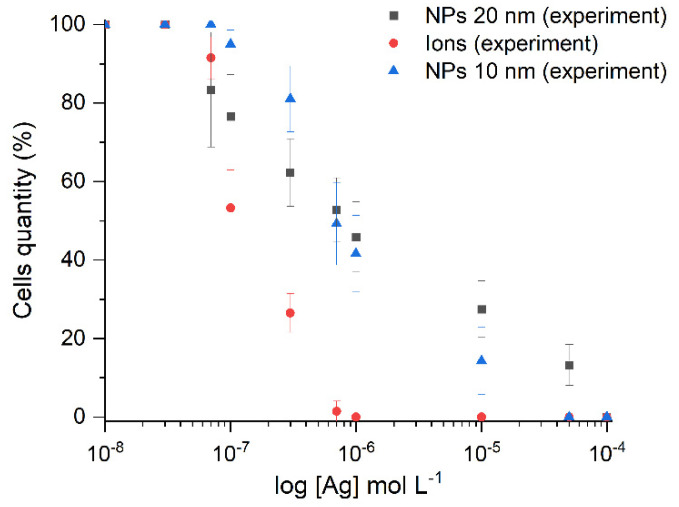
Quantity of cells: result of inhibition by various concentrations of silver.

Figure 6 illustrates the inhibitory activity of the carbonate hydrosols synthesized in this study against the growth of *Escherichia coli* bacterial cells for both Ag-NPs (10 nm) and Ag-Ag_2_O-NPs (20 nm). The results are comparable with the effect of Ag^+^ ions under identical conditions. The presented silver concentrations refer both to silver ions and to silver atoms in nanoparticles.

The half maximal inhibitory concentrations (IC_50_) were 1 × 10^−6^ mol L^−1^ for Ag-Ag_2_O-NPs (20 nm), 7 × 10^−7^ mol L^−1^ for Ag-NPs (10 nm), and 3 × 10^−7^ mol L^−1^ for Ag^+^ ions. The minimum inhibitory concentrations (MIC) were 1 × 10^−4^ mol L^−1^ for Ag-Ag_2_O-NPs (20 nm), 5 × 10^−5^ mol L^−1^ for Ag-NPs (10 nm), and 5 × 10^−5^ mol L^−1^ for the ions. Silver ions exhibited the highest antibacterial effect; the second most pronounced effect was found for 10 nm Ag-NPs, and 20 nm Ag-Ag_2_O-NPs were least efficient. However, 10 and 20 nm NPs showed rather similar effects. The effect of 20 nm NPs started at lower concentrations that that of 10 nm NPs. The pronounced effect of 10 nm NPs started in the concentration range of 3 × 10^−7^–1 × 10^−5^ mol L^−1^. The antibacterial effect of silver ions showed up at lower concentrations than that of nanoparticles. In the case of silver NPs, the concentration expresses the overall effect of the pristine NPs and the silver ions released from the NP surface upon partial oxidation. In Table 2, the data of measurement of the antibacterial action of the carbonate silver nanoparticles on the *Escherichia coli* cells are compared with the results obtained by other authors using other methods for silver nanoparticles of various sizes.

A systematic analysis of the data of Table 2 on the effect of silver on the vital activity of *Escherichia coli* indicates that the inhibiting concentrations are approximately 0.5–10 mg/L for silver ions and up to 5–100 mg/L for silver nanoparticles. However, some publications [47,60] indicate considerably higher concentrations, which may be caused by the use of larger particles and/or a different type of culture medium and experimental conditions. The MIC values obtained for carbonate nanoparticles are generally consistent with the data (5–100 mg/L) obtained by other authors. For example, MIC of the silver ions was approximately ~0.3 mg/L; that for Ag-NPs was ~5.4 mg/L; while the value for Ag-Ag_2_O-NPs increased to ~11 mg/L.

The set of the obtained data shows that the synthesized silver hydrosols have a high antibacterial activity; this useful feature is combined with other benefits related to the absence of impurities caused by the use of toxic reducing agents or stabilizers. The carbonate hydrosols are, in essence, environmentally benign materials containing silver nanoparticles and carbonic acid anions (mainly bicarbonate anions). The composition of the aqueous medium resembles that of natural freshwater.

#### 2.3.2. Determination of Morphology of the *Escherichia coli* Cells

##### Control

**Figure 7:**
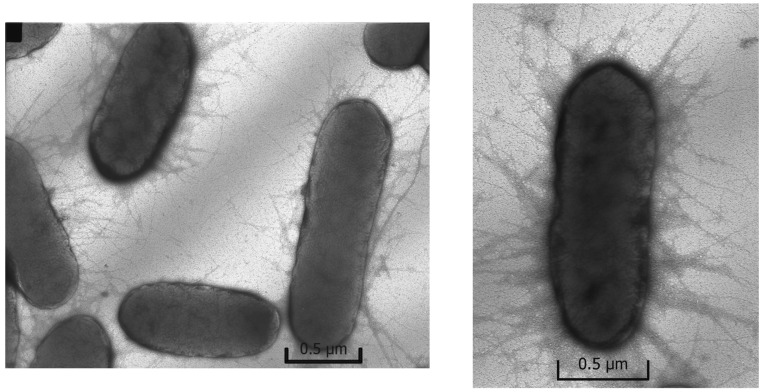
Photomicrograph of *Escherichia coli*, control.

The photomicrographs (Figure 7) of *Escherichia coli* grown in the control sample containing no silver, i.e., under favorable conditions, have an intact oval shape and have a lot of filaments and pili, which are responsible for the transfer of genetic material and adhesion.

##### Ions

**Figure 8:**
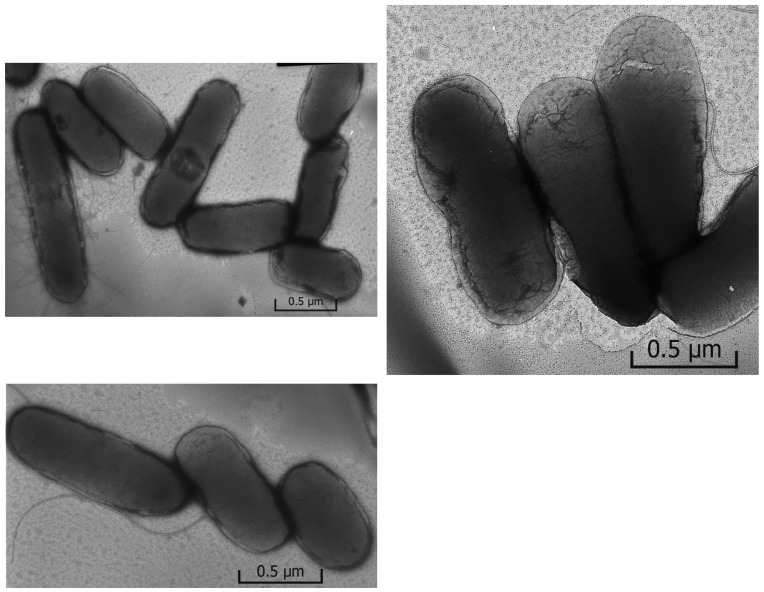
Photomicrographs of *Escherichia coli* grown in the presence of silver ions (1 × 10^−5^ mol L^−1^).

In the presence of ionic silver, the key morphological sign of its effect on the cells is almost complete disappearance of pili and filaments (Figure 8). The cells of *Escherichia coli* generally retain the smooth shape and are mainly not separated from one another. The cell damage is insignificant and, as shown below for the case of nanoparticles, it is not catastrophic. Generally, the cells do not undergo significant changes in the shape and preserve the membrane integrity.

##### Nanoparticles

**Figure 9:**
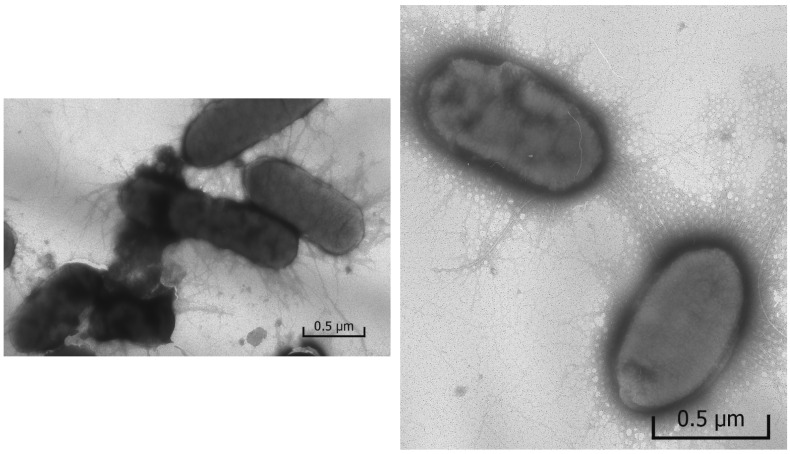
Photomicrographs of *Escherichia coli* grown in the presence of Ag-Ag_2_O-NPs (20 nm) (1 × 10^−5^ mol L^−1^).

The introduction of silver nanoparticles into the culture medium changes the cell morphology (Figure 9). When *E. coli* was grown in the presence of Ag-NPs (10 nm) or Ag-Ag_2_O-NPs (20 nm), the photomicrographs revealed deformation of the cell shape in comparison with the control, partial loss of pili, and pronounced destruction of cell membranes. The cell damage (destruction) is the most clear-cut indication of the effect of nanoparticles of both types. Many cells are not separated from one another, which probably accounts for inhibition of cell multiplication. Please note that in experiments carried out for 48 h, the oxidative dissolution of nanoparticles resulted in the release of approximately 25% (2.5 × 10^−6^ mol L^−1^) of the total amount of the metal into the solution as Ag^+^ ions. In other words, the catastrophic cell destruction is induced, first of all, by silver nanoparticles adsorbed on cell surface, because a larger amount of Ag^+^ ions (1 × 10^−5^ mol L^−1^) does not cause equal damage (compare Figure 8 and Figure 9).

Thus, it can be stated that ions and silver nanoparticles affect cells in different ways. It is beyond doubt that the effect of free Ag^+^ ions is the suppression of cells, which is morphologically manifested as the inhibition of development of pili and filaments. Only minor signs of cell destruction are present. The cell integrity is mainly retained. Nanoparticles with the same concentration of silver inhibit the development of pili and filaments to a markedly lesser extent than Ag^+^ ions. This is attributable to a markedly lower concentration of free Ag^+^ ions released to the solution upon the oxidative dissolution of nanoparticles (approximately 25% of 1 × 10^−5^ mol L^−1^). However, nanoparticles disrupt the cell integrity to a considerably higher extent, or rather damage the cells. The results imply the presence of a specific mechanism of toxicity of silver nanoparticles in comparison with the toxicity of Ag^+^ ions. Apart from the inhibition inherent in Ag^+^ ions, which is manifested as suppression of the development of *Escherichia coli* bacterial cells, they implement a specific contact mechanism of cell damage, resulting in cell destruction.

Perhaps, chemical degradation of the protective cell membrane of *Escherichia coli*, resulting in the cell destruction, takes place in the local site of contact as a result of oxidative dissolution of silver and formation of reactive oxygen species (O_2_^−•^/HO_2_^•^ and ^•^OH radicals and H_2_O_2_). The SEM and TEM data have demonstrated that the nanoparticle-treated *E. coli* cells are damaged, and pits are present in cell walls of the bacteria [61]. The silver nanoparticles are accumulated in the bacterial membrane. A membrane with this morphology exhibits a significant increase in permeability, which results in cell death.

#### 2.3.3. Change in the Cytoplasm Composition

**Figure 10:**
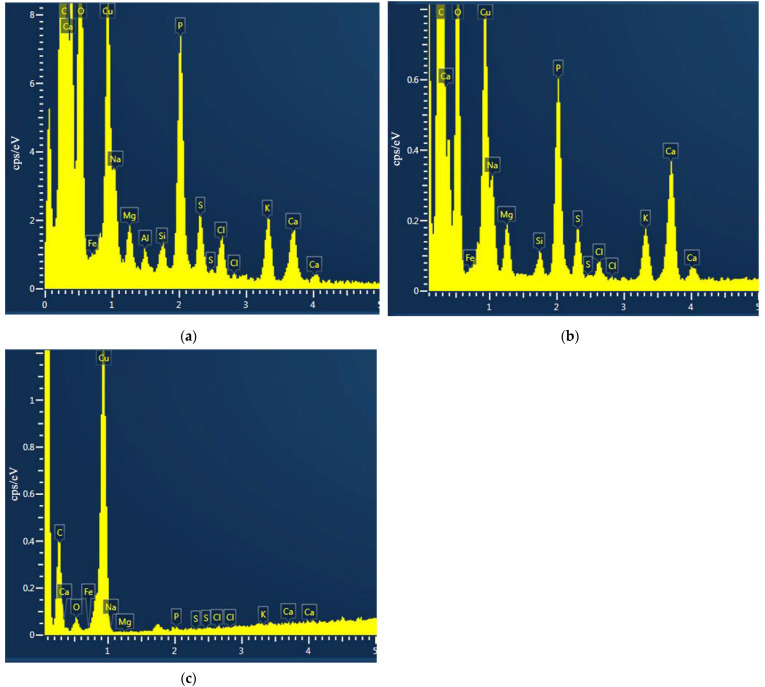
EDS spectrum of *Escherichia coli* cells: control (**a**); exposure with silver ions Ag^+^ (1 × 10^−5^ mol L^−1^) (**b**); exposure with Ag-Ag_2_O-NPs (1 × 10^−5^ mol L^−1^) (**c**).

An important indication of the vital activity of microorganisms is their chemical composition, particularly, the number of biogenic elements in the cells. When the *Escherichia coli* cells are grown in the presence of silver ions or silver NPs, this composition is markedly disrupted. The results presented in Figure 10 clearly show the decrease in the content of biogenic elements in the cells kept in the Adkins M culture medium in the presence of Ag^+^ ions or Ag-Ag_2_O-NPs (10^−5^ mol L^−1^). The losses of elements by the cells upon exposure with silver nanoparticles were: 99% for phosphorus, 98% for sulfur, 98% for potassium, and 98% for calcium. On exposure with silver ions, the loss of biogenic elements was somewhat less pronounced, but still very large. The loss of phosphorus was 91%, the loss of sulfur was 92% that of potassium was 86%, and that of calcium was 80%. The higher loss of elements in the case of exposure with nanoparticles is apparently attributable to the physical damage of the structure of bacterial cells and high cellular concentrations of reactive oxygen species (ROS). The results establish the pronounced inhibiting action of silver on the development of microflora. On the one hand, this attests to a high antibacterial activity of various silver species, but, on the other hand, this is a consequence of their toxicity.

The mechanism of interaction between the nanoparticles and the outer membrane of *E. coli* is not fully understood. The sticking of NPs to the cells is apparently accompanied by disruption of the structure of the bacterial membrane, causing structural changes and membrane degradation, which is followed by incorporation of silver nanoparticles into the membrane and, finally, cell death. TEM analysis (Figure 9) and the presence of elemental silver in the membranes of treated bacteria, detected in [61] using EDS, confirms the incorporation of silver nanoparticles into the membranes. The biocidal properties of silver nanoparticles are attributable to their high reactivity toward proteins, disruption of the cell wall and membrane structure, resulting in cell inhibition and death [43,45,62,63,64]. Thus, the antibacterial activity of nanoparticles is due to direct contact with the cell membrane, which disrupts its basic functions, and to the release of silver ions, which further contribute to the bactericidal effect [12,65,66,67].

### 2.4. Direct (Contact) and Indirect Mechanism of Action of Silver Nanoparticles on Escherichia coli

Extensive studies of the biocidal activity of silver nanoparticles, including the data of this work, make it possible to conditionally distinguish these two main factors of the nanoparticle effect on living cells. The indirect action of silver is related to the behavior of the Ag^+^ ion as a soft Lewis acid. The ion has a pronounced affinity for elements containing a lone pair of electrons. First of all, these elements are nitrogen, sulfur, and other elements that form biomolecules. Having attached to molecules containing these elements, the Ag^+^ ions disrupt the biochemical processes, thus suppressing the vital activity of cell structures. It is generally accepted that the adverse effect of silver ions is due to interaction with thiols and amino groups of proteins, nucleic acids, and cell membranes [46,68,69,70].

A distinctive feature of the toxic effect of silver nanoparticles on microorganisms is also their ability to induce oxidative stress. This is clearly manifested in the study of cell morphology as a process leading to cell destruction, which was observed in the present study. This mechanism of antibacterial (toxic) action of silver is mainly implemented via the direct contact of nanoparticles with bacteria. This condition is caused by high intracellular concentrations of reactive oxygen species (ROS), which include superoxide radical anions, hydroxyl radicals, and hydrogen peroxide molecules. ROS can directly react with membrane lipids, proteins, and DNA. A high concentration of ROS in bacterial cells may induce oxidative stress. The ability of Ag-NPs to cause oxidative stress in *Staphylococcus aureus*, *Escherichia coli*, and *Pseudomonas aeruginosa* was demonstrated previously [43].

The reactive oxygen species are apparently formed upon the electrochemical oxidative dissolution of nanoparticles. The reduction of oxygen (reaction (7)) involving transfer of four electrons to the O_2_ molecule to give four OH^−^ ions is actually the sum of several intermediate reactions, with their overall result being expressed by reaction (7). In reality, the reduction of O_2_ follows a complex stepwise mechanism including successive transfer of single electrons. First, the O_2_^−^/HO_2_ radical ion species and H_2_O_2_ molecules are formed [71]:

O_2_ + e^−^ → O_2_^−•^           *k* = 1.9 × 10^10^ mol L^−1^ s^−1^ (13)

O_2_^−•^ + H^+^ ↔ HO_2_^•^    pK = 4.7 (14)

HO_2_^•^ + HO_2_^•^ → H_2_O_2_ + O_2_      *k* = 1.6 × 10^6^ mol L^−1^ s^−1^, (15)

HO_2_^•^ + O_2_^−•^ → H_2_O_2_ + O_2_ + OH^−^    *k* = 9.5 × 10^7^ mol L^−1^ s^−1^, (16)

The O_2_^−•^/HO_2_^•^ radicals are highly chemically reactive and their lifetimes in water amount to a few microseconds, while the molecular compound H_2_O_2_ formed upon their recombination is relatively stable and plays an important role in oxidative and biochemical processes. It was shown [30] that H_2_O_2_ is formed upon the oxidative dissolution of silver nanoparticles. Hydrogen peroxide (0.42 μmol L^−1^) was detected in filtrates after a 3-h incubation of nAg (4.8 ± 1.6 nm size; concentration of 2 mg L^−1^) in air-saturated water (9.1 mg L^−1^ of dissolved O_2_, initial pH = 5.7).

The formation of ROS is not limited to the indicated reactions, but can also be accompanied by the formation of ^•^OH radicals via the following reactions [72,73,74,75]:

O_2_^−•^/HO_2_^•^ + H_2_O_2_ → ^•^OH + H_2_O + O_2_, *k* = 0.5–3.0 mol L^−1^ s^−1^, pH = 0.5–3.5 (17)

O_2_^−^ + H_2_O_2_ → ^•^OH + OH^−^ + O_2_, *k* = 0.13–2.25 mol L^−1^ s^−1^, pH = 5.4–8.0 (18)

Finally, one more important step is the reaction of O_2_^−•^/HO_2_^•^ with the products of CO_2_ hydrolysis in water giving peroxide and the CO_3_^−•^ radical anion with oxidative characteristics close to those of the ^•^OH radical [25,76].

O_2_^−•^/HO_2_^•^ + H_2_CO_3_/HCO_3_^−^/CO_3_^2−^ → HO_2_^−^ + CO_3_^−•^, *k* = 1–2 × 10^6^ L^−1^ mol s^−1^, pH 5.5 (19)

The formation of reactive oxygen species upon the oxidative dissolution of silver nanoparticles was indicated in several publications [77,78,79,80,81,82,83]. Danilczuk and co-workers [84] reported Ag-generated free radicals through the ESR study of Ag nanoparticles. An ESR study also detected radicals, thus confirming the formation of ^•^OH upon the reduction of H_2_O_2_ with silver nanoparticles in acid medium [85].

Ag_n_ + H_2_O_2_ → Ag_n−1_ + Ag^+^ + ^•^OH + OH^−^ (20)

The O_2_^−•^/HO_2_^•^ and ^•^OH radicals actively react with a variety of organic molecules, including components of biomolecules, to give organic radicals R^•^ [72,73,74,75]. The radicals R^•^ react with oxygen to give peroxide radicals ROO^•^. An important feature of ROO^•^ is the ability to initiate chain reactions.

Thus, silver nanoparticles can induce the formation of ROS, O_2_^−•^/HO_2_^•^ and ^•^OH radicals, and H_2_O_2_ molecules in the presence of oxygen during the oxidative dissolution of metal. In the local site of contact between the nanoparticle and the cell, high concentrations of reactive radicals induce the acute damage of the membrane, penetration of ions into the cell, and cell destruction. An important result of the study of nanoscale silver in aerated aqueous solutions and in natural freshwater is that the antibacterial (toxic) properties of silver are not limited to the release of toxic Ag^+^ ions, but also involve the formation of reactive oxygen species upon the oxidative dissolution of nanoparticles. This important circumstance should be taken into account in consideration of both toxic and antibacterial effects induced by silver nanoparticles. The peroxide H_2_O_2_ produced in the cells as a result of various biochemical processes in the presence of nanoparticles may serve as an additional source of ROS in biological processes.

## 3. Materials and Methods

### 3.1. Chemicals

Silver perchlorate monohydrate (AgClO_4_∙H_2_O, 99%, Sigma-Aldrich, Saint Louis, MO, USA) and potassium oxalate (K_2_C_2_O_4_, 99.9% special purity grade, Reakhim, Moscow, Russia) were used for silver hydrosol preparation.

Ammonium nitrate (NH_4_NO_3_, 99%), sodium bicarbonate (NaHCO_3_, 99%), sodium nitrate (NaNO_3_, 99%), glucose (99%), sodium sulfate (Na_2_SO_4_, 99%) and magnesium sulfate (MgSO_4_, 99%) purchased from LenReaktiv, Saint-Petersburg, Russia, yeast extract (99%, Research Center of Pharmacotherapy (NICF), Saint-Petersburg, Russia), and peptone (99%, Port Petrovsk, Kursk, Russia) were used for the preparation of liquid media.

### 3.2. Synthesis of Silver Nanoparticles

Preparation of a “pure” silver hydrosol composed of only silver NPs and carbonate ions is based on reduction of silver ions in solution with oxalate ions under pulsed UV irradiation. The source of silver ions, 3 × 10^−4^ mol L^−1^ aqueous silver perchlorate (AgClO_4_), was mixed with 1 × 10^−4^ mol L^−1^ potassium oxalate (K_2_C_2_O_4_) as the carbonate ion source (ionic stabilizing agent). Irradiation was carried out in air in a quartz cell (2 mL) with an optical path length of 5 mm. Solutions were irradiated by a pulse xenon lamp with the total radiation flux intensity *I*_UV_ = 6.0 × 10^20^ quantum/s (alpha-05, Melitta, Moscow, Russia). A detailed description of the synthesis and mechanism of formation of silver NPs was reported in our previous publications [22,23,24].

### 3.3. Physico-Chemical Characterization of Silver Nanoparticles

Optical spectra were measured using a Cary 100 Scan (Varian, Palo Alto, CA, USA) spectrophotometer, equipped with a thermostatic Peltier cell, at temperature *T* = 20 °C. The hydrodynamic size and *ζ*-potential of colloidal particles were determined by dynamic light scattering on Delsa Nano C (Beckman Coulter, Brea, CA, USA), the wavelength of the scattered laser radiation was *λ* = 658 nm. The sizes and polydispersity of the nanoparticles were determined with a JEM-2100 transmission electron microscope (TEM) (JEOL, Akishima, Tokyo, Japan) operating at an accelerating voltage of 200 kV. Support Formvar film coated with a “heavier” layer of carbon (Carbon Type-B 400 mesh, Cu. Ted Pella Inc., Redding, CA, USA) was used to make samples for TEM.

### 3.4. Determination of Dissolved Silver Ions

The ICP-MS method, Element 2 (Thermo-Finnigan, Bremen, Germany), was used to determine the concentration of dissolved Ag^+^ in the nutrient medium. To separate ions from nanoparticles, the nutrient medium with silver was centrifuged at 14,600 rpm for 30 min at Eppendorf Centrifuge 5424 (Eppendorf AG, Hamburg, Germany). After that, the optical spectra of the samples were measured. It was found that the bands of the optical spectrum characteristic of silver nanoparticles were absent, which indicates the removal of nanoparticles and the possibility of further determination of silver exclusively in ionic form.

### 3.5. Bacterial Strains and Cultivation of Bacteria

The Gram-negative bacterium *Escherichia coli* K-12 MG1655 from Russian National Collection of Industrial Microorganisms was chosen as the biological object to study the antimicrobial activity of produced silver NPs.

Cells were grown in the early reported liquid low mineralized Adkins medium [86] with few changes (Adkins M: NH_4_NO_3_, 1 mg/L; NaHCO_3_, 0.4 mg/L; NaNO_3_, 1 mg/L; glucose, 2 mg/L; yeast extract, 1 mg/L; peptone, 1 mg/L) at *T* = 25 °C for 48 h.

After that the cell suspension was used as an inoculum to study the antibacterial action of silver in the experiments described below.

### 3.6. Inhibition of the Bacterial Cell Growth

The completely grown (as described above) bacterial solution containing *E. coli* was inoculated into the same culture medium (1:10 ratio). The volume of each sample was 50 mL. In total, three samples were prepared: (1) cells grown without silver (control), (2) cells grown with NPs, and (3) cells grown with silver ions. For silver-containing samples, either the hydrosol containing nanoparticles or an aqueous solution of silver ions was added to the medium simultaneously with the cells. The silver concentration varied from 1 × 10^−8^ to 1 × 10^−4^ mol L^−1^. The exposure time was 48 h at *T* = 25 °C. Each sample was prepared in triplicate. Then, after thorough mixing, a drop (10 µL) was taken with a syringe from each sample; the drops were deposited on a slide glass, and the number of cells in the field of vision was counted. The immersion method was used for microscopic examination on a MicroOptix MX300 microscope (West Medica, Wiener Neudorf, Austria). The number of cells in the control was taken to be 100%. In the silver-containing samples, the cell count was either the same (at low concentrations) or lower; the cell count was calculated relative to the control (relative cell count = $\frac{\mathrm{cell}\;\mathrm{count}\;\mathrm{in}\;\mathrm{the}\;\mathrm{sample}}{\mathrm{cell}\;\mathrm{count}\;\mathrm{in}\;\mathrm{the}\;\mathrm{control}}\times 100\%$).

The IC_50_, the half maximal inhibitory concentration, is the concentration required to inhibit 50% of the bacterial growth. It was determined by mathematical calculations in program OriginPro (nonlinear curve fit “growth/sigmoidal”). MIC, minimal inhibitory concentration, is the lowest concentration of antimicrobial agent that completely inhibit cell growth. It was determined as the concentration at which the number of cells was the same as during inoculation (i.e., no growth was observed).

### 3.7. Effect of AgNPs on the E. coli Investigated by TEM and EDS

The effect of silver on *E. coli* cells was studied by transmission electron microscopy (TEM) and energy-dispersive X-ray spectroscopy (EDS). A JEM 1400 microscope (JEOL, Akishima, Tokyo, Japan) was used for TEM and an INCA Energy TEM 350 instrument (Oxford Instruments, Abingdon-on-Thames, Great Britain) was used for EDS. The cells were grown in the same way as described above. The concentration of silver was 1 × 10^−5^ mol L^−1^.

## 4. Conclusions

Thus, silver as spherical nanoparticles stabilized by carbonate ions has a suppressing effect on *Escherichia coli*. It is noteworthy that when silver hydrosols are obtained by photochemical reduction, only silver acts directly on the bacterial cells, since there are no toxic reducing agents, stabilizers, or products of their decomposition. Silver ions and nanoparticles inhibit the growth of *Escherichia coli* when present in concentrations of ~1 × 10^−6^–1 × 10^−4^ mol L^−1^. The nanoparticles obtained in deaerated and aerated solutions (10 nm Ag-NPs and 20 nm Ag-Ag_2_O-NPs, respectively) remain stable in air for several months. Moreover, they are stable for 1–3 weeks in the Adkins M medium, which is important for investigation of their biocidal properties. The mechanism of antibacterial (toxic) action of silver nanoparticles on the microflora was proposed and justified. The mechanism includes indirect suppressive action of Ag^+^ ions, released upon the oxidative dissolution of the metal, and direct (contact) action of nanoparticle on the bacterial cell, resulting in a change of the cell shape and cell destruction.

The ability of nanoparticles to be slowly oxidized in aerated aqueous solutions and to retain their bactericidal properties constitute the useful effect of the dispersed form of silver for the application as an efficient and controllable biocidal material. On the other hand, the same feature of nanoscale silver is a source of hazardous toxic action of silver when it enters the environment. The prolonged oxidation and the formation of Ag^+^ ions continues with time, which also makes an additional bactericidal effect. Thus, nanosilver is a bactericidal material of prolonged action.

## Figures and Tables

**Table 1:** Characteristics of silver nanoparticles.

Nanoparticle	*λ*_max,_ nm	*W*_1/2_, nm	*d*_TEM_, nm	*d*_DLS_, nm	*ζ*, mV	Hydrosol Stability, *τ*_1/2_, Months
Ag-NP	386	35	10.1 ± 2.8	12.3 ± 2.5	−68.3	2–3
Ag-Ag_2_O-NP	408	86	22.3 ± 4.2	24.1 ± 4.0	−67.4	>6

**Table 2:** Antibacterial characteristics of Ag^+^ and nanoparticles against *Escherichia coli* cells.

Type	Size, nm	Surface Functionalization	Effect	Medium	Reference
Ag^+^	-	-	MIC = 0.3 mg L^−1^, IC_50_ = 0.03 mg L^−1^	Adkins M	This work
Ag-Ag_2_O nanoparticles	22 ± 3	Carbonate	MIC = 11 mg L^−1^, IC_50_ = 0.11 mg L^−1^	Adkins M	This work
Ag nanoparticles	10 ± 2	Carbonate	MIC = 5.4 mg L^−1^, IC_50_ = 0.07 mg L^−1^	Adkins M	This work
AgCl nanoparticles	250	Unfunctionalized	0.5 mg L^−1^ inhibits bacterial growth by 66 ± 6%	BBL™ containing 5 g/L Gelysate™ peptone and 3 g/L beef extract, pH = 6.9 ± 0.2	[46]
Ag nanoparticles	14–16	PVA	0.5 mg L^−1^ inhibits bacterial growth by 55 ± 8%	BBL™ containing 5 g/L Gelysate™ peptone and 3 g/L beef extract, pH = 6.9 ± 0.2	[46]
Ag^+^	-	-	0.5 mg L^−1^ inhibits bacterial growth by 100%	BBL™ containing 5 g/L Gelysate™ peptone and 3 g/L beef extract, pH = 6.9 ± 0.2	[46]
Ag nanoparticles (octahedral)	194 ± 50	PVP	MIC = 50 mg L^−1^	LB medium	[47]
Ag nanoparticles (spherical)	195 ± 50	Citrate	MIC not achieved, IC_50_ = 1000 mg L^−1^	LB medium	[47]
Ag nanoparticles	7	Gallic acid	MIC = 6.25 mg L^−1^	Mueller–Hinton broth	[48]
Ag nanoparticles	29	Gallic acid	MIC = 13.02 mg L^−1^	Mueller–Hinton broth	[48]
Ag nanoparticles	89	Gallic acid	MIC = 11.79 mg L^−1^	Mueller–Hinton broth	[48]
Ag nanoparticles	12.2	Citrate	MIC = 13.8 mg L^−1^	Cation-adjusted Mueller–Hinton broth (CA-MHB)	[49]
Ag nanoparticles	10.2 ± 2.3	Citrate	IC_50_ = 5 mg L^−1^ MIC = 15 mg L^−1^	2 mM NaHCO_3_	[50]
Ag nanoparticles	10.2 ± 2.3	Citrate	IC_50_ = 5 mg L^−1^ MIC = 15 mg L^−1^	2 mM NaHCO_3_	[50]
Ag nanoparticles	9.9 ± 2.0	Mercaptopropionic sulfonic acid	MIC = 15 mg L^−1^	2 mM NaHCO_3_	[50]
Ag nanoparticles	16.6 (6.5–43.8)	Not reported	IC_50_ = 1.56 mg L^−1^ IC_90_ = 6.25 mg L^−1^ MIC = 12.5 mg L^−1^	DMEM supplemented with l-glutamine (4 mM), penicillin (100 units/mL), streptomycin (100 μg/mL) and 10% (*v*/*v*) heat inactivated fetal bovine serum	[51]
Ag nanoparticles	10	Citrate	30 mg L^−1^ inhibit bacterial growth; EC_50_ (CFU assay) = 3.2–4.2 mg L^−1^; EC_50_ (LTP assay, 0 h) > 0.25 mg L^−1^; EC_50_ (LTP assay, 1 h) = 0.06–0.09 mg L^−1^; EC_50_ (LTP assay, 2 h) < 0.025 mg L^−1^;	LB medium	[52]
Ag_2_S nanoparticles	9 ± 3.5	Unfunctionalized	Not toxic at 150 mg L^−1^	RPMI medium supplemented with 0.2 mM l-glutamine, 100 μg mL^−1^ penicillin, 100 μg mL^−1^ streptomycin and 10% FBS	[53]
Ag^+^	-	-	MIC = 3.5 mg L^−1^ for 10^3^ cells; MBC = 3.5–5 mg L^−1^	LB medium	[54]
Ag^+^	-	-	MIC = 0.5–1 mg L^−1^ for 10^3^ cells, MBC = 0.5–1.25 mg L^−1^	RPMI/FCS	[54]
Ag nanoparticles	75 ± 20	PVP	MBC = 12.5–20 mg L^−1^ for 10^3^ cells	RPMI/FCS	[54]
Ag nanoparticles	11.3 (3–40)	Laser ablation	MIC = 110 ± 16 mg L^−1^ (microdillution assay); MIC = 73 ± 11 mg L^−1^ (optical density assay);	Nutritionally impoverished LB (5.0 g of tryptone, 2.5 g of yeast extract, and 5.0 g of NaCl per 1 L)	[55]
AgCl nanoparticles	3	Unfunctionalized	MIC = 2 mg L^−1^	LB	[56]
Ag nanoparticles	10.8 ± 4.2	Bis-2-ethylhexyl sulfosuccinate (AOT)	MBC = 0.3 mg L^−1^	LB (solid, with agar)	[57]
Ag nanoparticles	10.8 ± 4.3	Cetyltrimethyl-ammonium bromide (CTAB)	MBC = 0.2 mg L^−1^	LB (solid, with agar)	[57]
Ag nanoparticles	13.5 ± 7.1	Poly-L-lysine (PLL)	MBC = 0.2 mg L^−1^	LB (solid, with agar)	[57]
Ag nanoparticles	15.2 ± 6.9	Polysorbate 80 (Tween 80)	MBC = 0.5 mg L^−1^	LB (solid, with agar)	[57]
Ag^+^	-	-	IC_50_ = 7 mg L^−1^ 12 h) MIC = 10 mg L^−1^ (12 h)	LB medium containing ampicillin (100 μg mL^−1^)	[58]
Ag nanoparticles	39.5 ± 10.7	PVP	IC_50_ = 4 mg L^−1^ 12 h) MIC = 30.2 mg L^−1^ (12 h)	LB medium containing ampicillin (100 μg mL^−1^)	[59]
Ag nanoparticles	4.65 ± 0.5	Citrate	MIC = 5.59 mg L^−1^	LB	[59]
Ag nanoparticles	38.5	PVP	MIC = 700 mg L^−1^	DMEM containing 10% fetal calf serum and 1% penicillin G-streptomycin	[60]

## Data Availability

Not applicable.
